# Feasibility and effectiveness of an online mindfulness meditation program for medical students

**Published:** 2018-11-12

**Authors:** Marlon Danilewitz, Diana Koszycki, Heather Maclean, Millaray Sanchez-Campos, Carol Gonsalves, Douglas Archibald, Jacques Bradwejn

**Affiliations:** 1 University of British Columbia, British Columbia, Canada; 2 Univeristy of Ottawa, Ontario, Canada; 3 Institut du Savoir – l’Hôpital Montfort, Ontario, Canada; 4 University of Ottawa Brain and Mind Research Institute, Ontario, Canada; 5 The Ottawa Hospital, Ontario, Canada; 6 Bruyère Research Institute, Ontario, Canada

## Abstract

**Background:**

The need to incorporate tools to promote medical student wellness in medical education is underscored by the concerning rates of psychological distress among them. The objective of this prospective cohort study was to obtain preliminary data on the feasibility and effectiveness of an online mindfulness intervention for medical student wellness.

**Methods:**

A convenience sample of 52 medical students consented to participate in this study. Feasibility was assessed by ease of recruitment, number of modules completed, satisfaction with the program, and adherence to a regular meditation practice. Participants completed the Maslach Burnout Inventory, the Jefferson Scale of Empathy-medical student version, the Five Face of Mindfulness Questionnaire-short form, and the Self Compassion Scale-short form pre and post intervention.

**Results:**

The convenience sample was recruited within a two-month period. Forty-five participants completed at least one of seven modules. Descriptive statistics (mean±standard deviation) revealed that the mean number of modules completed was 4.85±2.7. Mean satisfaction with the modules was 7.07±1.1 out of 10. Adherence to a regular formal meditation practice was poor; the average amount of formal meditation practice per module was 34.14±27.44 minutes. Self-compassion and the “observe and describe” facets of mindfulness practice significantly statistically increased from baseline, but no such change was observed for levels of burnout and empathy.

**Conclusion:**

The present study indicates that an online mindfulness meditation program may be of interest to medical students. The results did not provide any evidence that the program was effective but we believe further research and development is warranted.

## Introduction

The road to become a physician presents medical students with a constellation of stressors, culminating in high rates of psychological morbidity.^1,2^ According to a recent meta-analysis that included 195 studies involving 129,123 medical students, the prevalence of depression was 27.2% and that of suicidal ideation was 11.1%.^3^ Increased levels of distress, in turn, have been linked to poor academic performance and increased dropout rates from medical school.^4^ Moreover, physician distress and depression have been shown to correlate with decreased patient quality of care as a result of reduced empathy and increased medical errors.^5,6^ The inability to manage stress and regulate negative emotions can compromise the ability to deliver compassionate medical care.^7^

Over the last several years, mindfulness meditation has emerged as a popular self-regulation strategy to manage stress and improve emotional well-being. The most well-known and researched mindfulness-based program is Mindfulness-Based Stress Reduction (MBSR) developed by Kabat-Zinn.^8^ This eight-week group program includes formal training in focused attention and open monitoring of moment-to-moment experiences, as well as informal practices to cultivate mindfulness in daily routine activities. Training in focused attention and awareness, coupled with an attitude of nonjudgment, openness, and acceptance, fosters greater emotional and cognitive flexibility, which is presumed to underlie the beneficial effects of mindfulness on psychological well-being.^9^ Neuroimaging studies suggest mindfulness meditation can rewire how the brain responds to stress and improves working memory and executive functioning.^10^

Mindfulness meditation has been shown to have a therapeutic benefit in reducing stress, anxiety and depression.^11,12^ In a review of the literature of MBSR for healthy individuals, Chiesa and colleagues^13^ concluded that MBSR reduces ruminative thinking and trait anxiety and enhances empathy and self-compassion. A small number of studies have examined the impact of mindfulness programs in medical student populations. One study of 302 second-year medical students found that mood disturbances decreased with statistical significance following ten-week training in mindfulness.^7^ In a randomized controlled trial of an eight-week meditation program for medical and premedical students, a significant reduction in state and trait anxiety was observed in participants assigned to the meditation program.^12^ In a large open trial of 239 medical students, an eight-session wellness program that included mindfulness meditation significantly reduced depression and hostility and improved the psychological domain of the WHO Quality of Life scale.^14^ Mindfulness meditation has also been shown to increase empathy and compassion in health professional populations.^15,16^ In a feasibility study of a peer-led mindfulness program for medical students, decreased levels of stress and enhanced mindfulness, self-compassion, and altruism were observed from pre to post-study.^17^

Although previous findings are encouraging, there remains a paucity of well-designed studies of mindfulness-based interventions for medical student wellness. This limitation may actually stem from curricular and practical issues. Medical students face extreme time pressures given demanding medical curricula, rendering participation in weekly group longitudinal programs exceedingly challenging. While a number of medical schools have begun to integrate elements of mindfulness into the formal curricula, these attempts, while exemplary, are often limited by curricular constraints. The eight-week standard curriculum is often abridged or sessions are interspersed or non-contiguous. Moreover, little is known about whether students actually adopt a regular meditation practice following formal lectures/tutorials on mindfulness. The largely elective nature of the majority of reported interventions is another limitation, as a highly self-selected group of participants is likely involved. As noted earlier, in-person mindfulness programs report low rates of adherence and high dropout rates, suggesting that standard mindfulness programs may not meet the availability or needs of medical students.

Online mental health interventions are the newest frontier, offering potential in both primary and secondary prevention of mental illness, treatment of mental illness, as well as an even wider benefit in the area of stress management. Online mental health programs boast ease of access and low cost and obviate concerns for finding both a skilled and proximate meditation teacher. Online psychological therapy and psychoeducation have demonstrated comparable efficacy to face-to-face therapy for a number of conditions, including stress, depression and anxiety.^18^ Research evaluating online mindfulness-based interventions for psychological disorders and stress reduction is still in its infancy. Morledge and colleagues conducted a randomized controlled trial of an online mindfulness intervention for stress management and found the online intervention produced a statistically significant benefit in levels of perceived stress, mindfulness, self-transcendence, and psychological wellbeing.^19^

With growing popularity of web-based learning tools in medical education an online mindfulness meditation program might be a viable strategy to address some of the limitations of traditional mindfulness programs for medical students. Kemper et al.^20^ recently conducted a small pilot study with seven medical students involving a hybrid of an online mindfulness intervention with peer facilitation that showed evidence of feasibility. The advantages of an online mindfulness program are numerous: it can reach a large audience of medical students in a cost-effective manner; it can be accessed from almost any setting, eliminating attendance difficulties; it is available around the clock, making it flexible for students with many daily commitments; it allows students to work at their own pace; and it can be delivered privately and anonymously, reducing student concerns about stigma accessing face-to-face wellness programs. Similarly, an online program would further access to students studying at distributed sites, on rural rotations, or on electives. Moreover, an online program could allow students to return to modules after going through the online curriculum in its intended course, as well as allow them to track their meditation practice and the benefit that their practice may have on wellbeing. All of these added features might bolster adherence and completion rates. Finally, an online program can act as a stand-alone option for medical students or as an adjunct to existing face-to-face mindfulness or other wellness programs.

The primary aim of the current study was to obtain preliminary data of whether an online *Mindfulness-Based Program for Medical Student Wellness* (“MIND-MED”) is feasible. The secondary aim was to obtain preliminary data on its effects on medical student burnout, empathy, mindfulness, and self-compassion.

## Methods

### Design

The study adopted a prospective pilot cohort design. All participants who consented to participate in the study were given access to the online program.

### Participants

Participants were medical students at the University of Ottawa who were recruited via email invitation sent out by the medical school student council president. In total, three email invitations were sent over a one-month period to all students enrolled in the medical program, approximately 600. A recruitment goal of 50 students was selected for the study. This sample size was chosen a priori as it is considered adequate for testing the feasibility of a new intervention.^21^ In total, 52 students were enrolled in the study on a first-come first-served basis. The inclusion criteria to participate were 1) provision of informed consent; and 2) understanding that the program is not a “therapy” to be used in replacement for professional care for psychological and health issues. There were no exclusion criteria. Participants received a $10 gift certificate at each assessment time point, to a maximum of $20, and those who completed all seven modules of the program received a medical school performance record (MSPR) credit through the Wellness Student Interest Group. The MSPR is a document prepared by the University of Ottawa Faculty of Medicine for all medical students. The document outlines each medical student’s involvement in department related activities over their years of study. There is no minimum or maximum number of activities medical students must participate in. Ultimately, the MSPR document is submitted as part of a medical student’s residency application to highlight their overall participation in medical school related activities. This study received ethics approval from the Ottawa Health Science Network Research Ethics Board (file # H10-14-19B).

### Mindfulness Intervention

The MIND-MED program was developed by a team of medical students and clinical researchers with previous experience evaluating mindfulness interventions in medical school at the University of Ottawa. Two web developers assisted in building the program. The web developers were consulted through an iterative design process and a mock-up prototype was developed. Once the mock-up prototype was approved by the research team the web developer translated the mock-up into the fully functional program for pilot testing.

MIND-MED incorporates seven online modules that address pillars of mindfulness practice as well as themes specifically relating to the experience of medical students. The modules included video material followed by a meditation practice and were completed sequentially as each module builds upon material covered in earlier modules. Each module was 25 to 35 minutes in duration. Participants were encouraged to engage in daily formal (e.g., body scan, sitting meditation, mindful yoga, walking meditation) and informal (e.g., mindful eating, mindfulness of daily activities) meditation practices. The website included downloadable audio recordings of different meditation practices of varying durations (e.g., 15- or 30-minute body scan meditation) and students were asked to record how many minutes they engaged in mindfulness practice each week. For mindful yoga, both audio and video recordings of yoga postures were provided. Relevant reading material about mindfulness and student wellbeing were posted on the website as well as links to other websites that contained information about mindfulness. Reminder emails were sent each week to encourage participants to complete the module if they had not done so. However, as this was a purely self-administered program, no email or telephone support was provided. The duration of the intervention based on participant rate of progression through the modules ranged from a minimum of seven weeks to a maximum of four months.

### Outcomes

Feasibility criteria included ease of recruitment, program completion rate, adherence to a regular meditation practice, and program satisfaction. Program satisfaction was evaluated globally on a 0 (not at all satisfied) to 10 (extremely satisfied) scale. A 5-point Likert scale was used to evaluate participants’ perception of module relevance to medical students (Irrelevant to Very Relevant), module length (extremely short to extremely long), and usefulness of supporting module materials (extremely unhelpful to very helpful). Open-ended qualitative feedback regarding the modules was elicited at the end of each module for eventual refinement of the program. The following self-report measures were completed online by participants at study enrollment and after completion of the final module or at the point at which a participant dropped out of the study:

#### Maslach Burnout Inventory (MBI)^22^

The MBI is a 22-item scale with three primary factors: emotional exhaustion (feelings of being emotionally over extended and exhausted by one’s work); depersonalization (an unfeeling and impersonal response toward recipients of one’s care); and personal accomplishment, (feelings of competency and successful achievement in one’s work). Items are rated on a 7-point Likert-type scale. High scores on emotional exhaustion (≥ 27) and depersonalization (≥10) and low scores on personal accomplishment (≤33) are indicative of burnout. The scale has satisfactory psychometric properties including good internal consistency for each of the three subscales (α range from .74 to .89).^22^ In the current study, participants were identified as being “burnt out” if they scored high on both the Emotional Exhaustion and Depersonalization subscales. The Personal Accomplishment subscale was not included in our definition of burnout as this subscale is thought to measure a dimension that is distinct from the other MBI subscales.

#### Jefferson Scale of Empathy-Medical Students (JSE-S)^23^

The JSE-S is a 20-item Likert-type self-report questionnaire assessing health care professional attitudes towards empathy in patient care settings. Items are rated on a 7-point scale, with higher scores indicating higher levels of empathy. The scale measures three dimensions of empathy (perspective taking, compassionate care, and grasping the patient’s experience). The average empathy score is 114.3 ± 10.4 for US medical students. A score ≤95 for men and ≤100 for women indicates low levels of empathy, and a score ≥127 for men and ≥129 for women reflects high levels of empathy.^24^ The scale has excellent psychometric properties including high internal consistency for medical residents (α = .87) and medical students (α= .89).^23^

#### Five Facet Mindfulness Questionnaire-Short Form (FFMQ-SF)^25^

The FFMQ-SF is a 24-item scale that assesses five facets of mindfulness: observing (“When I’m walking, I deliberately notice the sensations of my body moving”), describing (“I’m good at finding words to describe my feelings”), acting with awareness (reverse item: “When I do things, my mind wanders off and I’m easily distracted”), non-judging of inner experience (reverse item: “I tell myself I shouldn’t be feeling the way I’m feeling”), and non-reactivity to inner experience (“I watch my feelings without getting lost in them”). Items are rated on a 5-point Likert-type scale with higher scores on each facet indicating higher levels of mindfulness. The scale has excellent psychometric properties including high internal consistency (αs = .75 to .91).^25^

#### Self-Compassion Scale-Short Form (SCS-SF)^26^

The SCS-SF is 12-item scale that captures how respondents perceive their actions toward themselves in difficult times (e.g., “When times are really difficult, I tend to be tough on myself”). Items are rated using a 5-point Likert-type scale with higher scores indicating higher levels of self-compassion. The scale measures three main components of self-compassion: Self-Kindness versus Self-Judgment, Common Humanity versus Isolation, and Mindfulness versus Over-Identification. The SCS-SF has demonstrated good internal consistency (Cronbach’s alpha ≥ .86) and a strong correlation with the long form SCS (r ≥ .97).^26^

### Statistical methods

Data were analyzed with SPSS Version 23. Descriptive statistics (percentages, means± standard deviations) were used to examine participant characteristics and feasibility outcomes. We used *t*-tests and Chi-square tests to compare participants who started the program versus those who did not on demographics, medical school year, and baseline self-report measures. Pre- to post-intervention changes in self-report measures were analyzed with linear mixed models. Linear mixed models use the full data set and can accommodate missing values without the need for imputation. The models were estimated by means of maximum likelihood and an unstructured covariance structure to account for correlations among the repeated measures; mean scores are reported as estimated marginal means with standard errors. Bivariate correlations were used to explore associations between feasibility outcomes (e.g., number of modules completed, compliance with mindfulness practice) and self-report questionnaires. Statistical significance was set at p<.05.

## Results

### Sample characteristics

Of the 52 students who enrolled in the study, 36 (69.2%) were female. The mean age of participants was 23.8±2.7 years (range: 20 to 37 years). Most students (79.9%) were in their first (n=21) or second (n=20) year of medical school. Ten students were in their third year and one was in their fourth year.

### Feasibility

We reached our target sample within approximately two months of active recruitment. Of the students who enrolled in the study, 45 (86.5%) completed at least one module. There was no difference between participants who did not start the program versus those who completed at least one module on demographics, medical school year, or baseline self-report measures. Of those who started the program, ten (22.2%) completed one to three modules, seven (16.7%) completed four to six modules, and 28 (66.7%) completed all seven modules; the mean number of modules completed was 4.85±2.7 out of seven. We found no statistically significant associations between the numbers of completed modules and participants’ gender, age, medical school year, or baseline scores on self-report measures. The mean reported number of minutes of formal meditation per module was 34.14 ±27.44 minutes (range: 0-140 minutes) and for informal meditation 34.62 ±24.2 minutes (range: 0-118.4 minutes). Although few participants (n=6; 13.3%) provided specific feedback regarding challenges with establishing a home meditation practice, the main reasons cited for not practicing included lack of time (e.g., “I am struggling to find the time to meditate”), difficulty remembering to meditate daily (e.g., “It is difficult to remember to do formal meditation everyday”) and feeling too stressed to meditate (e.g., “I feel too stressed to meditate. I understand it is counterintuitive. I will make an effort.”). With regards to program acceptability, the mean satisfaction score was 7.07±1.1 out of 10 (range: 4 to 9.7). No significant association was found between program satisfaction and the number of modules completed (r=.28, p=.07). Table 1 provides descriptive data of students’ feedback regarding module relevance, length, and usefulness of supportive materials.

**Table 1:** Participant Feedback about the Program Modules

	Module 1 Burnout in medicine	Module 2 The science of mindfulness	Module 3 Barriers to Mindfulness in Medical School: Being Mindful in Everyday Life and Medical School	Module 4 Common Questions and Troubleshooting	Module 5 Self Acceptance: Dealing with Perfectionism Relating to Our Judging Mind	Module 6 Relating to our judging mind: Loving Kindness Meditation	Module 7 Moving Beyond the Program
**Relevance to Medical Students**							
Very relevant	24.3%	32.7%	37.8%	23.5%	45.1%	25.8%	39.3%
Moderately relevant	75.7%	57.9%	54.1%	70.6%	45.1%	58.1%	46.4%
Neither relevant nor irrelevant	-	15.8%	8.1%	3.0%	9%	16.1%	10.7%
Somewhat irrelevant	-	-	-	-	-	-	-
Irrelevant	-	-	-	-	-	-	-
**Length**							
Acceptable	62.2%	62.2%	48.6%	60.0%	69.7%	77.4%	82.6%
Long	18.9%	24.3%	32.4%	28.6%	24.2%	19.3%	3.4%
Extremely long	8.1%	8.1%	13.5%	11.4%	3.0%	3.2%	3.4%
Short	2.7%	5.4%	2.7%	-	-	-	10.3%
Extremely short	-	-	-	-	-	-	-
**Usefulness of Supporting Materials**							
Very helpful	27%	22.8%	18.9%	25.7%	42.4%	38.7%	53.6%
Moderately helpful	54%	62.8%	64.9%	51.4%	45.4%	38.7%	32.1%
Neither helpful nor unhelpful	18.9%	14.3%	16.2%	22.9%	12.1%	22.6%	14.3%
Somewhat helpful	-	-	-	-	-	-	-
Extremely helpful	-	-	-	-	-	-	-

### Preliminary efficacy

Post-intervention assessments were completed by 37 of the 52 (71.2%) students who enrolled in the study. Completion rates varied according to year of medical school, with third and fourth year students having lower rates of completion (36.4%) than first (71.4%) and second (90%) year students (Fisher’s exact p=.008).

Table 2 displays the estimated marginal means (with standard errors) for pre- and post-intervention self-report measures and the estimated mean change from baseline (with 95% confidence intervals). Results failed to show a statistically significant effect of the online intervention on the burnout (MBI) subscales Emotional Exhaustion (F=4.02, df=1, 46.10, p=.051), Depersonalization (F=0.14, df=1, 43.59, p=.71) and Personal Achievement (F=0.37, df=1, 43.02, p=.55). The proportion of participants who met criteria for burnout was comparable across time points: 25.5% (13/51) at baseline and 27% (10/37) at post-intervention.

**Table 2:** Estimated marginal mean (±standard error) for pre- and post-intervention self-report measures and estimated mean pre- to post-intervention change (95% confidence intervals)

Scale	Pre-intervention	Post-intervention	Change (95% CI)	P value
**Maslach Burnout Inventory**				
Emotional exhaustion	27.6±1.3	24.1±1.4	-3.49 (-6.99, 0.02)	.051
Depersonalization	9.6±0.6	9.3±0.7	-0.31 (-1.97, 1.35)	.71
Personal accomplishment	42.5±0.9	43.5±1.3	0.89 (-2.07, 3.85)	.55
**Jefferson Empathy Scale-Student version**	113.5±1.7	115.7±0.2	2.21 (-0.10, 4.53)	.06
**Self-Compassion Scale**	35.0±1.2	39.3±1.3	4.33 (1.93, 6.72)	.001
**Five Facet Mindfulness Questionnaire**				
Non-judge	14±0.5	15.1±0.6	1.02 (-0.24, 2.27)	.11
Act with awareness	14.9±0.6	16.1±0.7	1.18 (-0.32, 2.68)	.12
Describe	16.7±0.5	18.4±0.6	1.71 (0.94, 2.47)	≤.001
Observe	12.9±0.5	15.1±0.5	2.27 (1.29, 3.24)	≤.001
Non-react	15.2±0.5	16.0±0.5	0.87 (-0.32, 2.05)	0.15

Regarding self-report empathy, baseline JSE-S scores were within the average range,^24^ with 11.5% (n = 6/52) of participants meeting threshold for low empathy and 11.5% (n= 6/52) meeting threshold for high empathy. Results failed to show a significant effect of the online program on JSE-S scores (F=3.78, df=1, 35.38, p=.06). At post-intervention, 10% (4/37) of participants met threshold for low empathy and 18.9% (7/37) met threshold for high empathy.

For the measure of mindfulness (FFMQ) there was a significant increase from baseline for the facet observe (F=22.16, df=1, 39.82, p<.001) and describe (F=20.63, df=1, 35.36, p<.001), but not for the facets act with awareness (F=2.54, df=1, 38.21, p=.12), non-judge (F=2.70, df=1, 35.73, p=.11), and non-react (F=2.18, df=1, 42.41, p=.15). Ratings of self-compassion (SCS-SF) also significantly increased from baseline (F=13.41, df=1, 37.15, p=.001).

Bivariate correlations between the number of modules completed, minutes of meditation and change from baseline in self-report questionnaires only yielded a significant correlation between pre- to post-intervention change in the non-judge facet of the FFMQ and minutes of informal meditation (r=.39, p=.024).

## Discussion

This study evaluated the feasibility and efficacy of an online mindfulness meditation program for medical students. With regards to feasibility, our recruitment goal was easily achieved, with 52 participants enrolling in the study over two months. Completion of the online post-study assessments was good, although this varied according to medical school year, with third and fourth year students reporting the lowest completion rate. Nevertheless, the 71% completion rate of post-study questionnaires exceeds that of other investigations of online mindfulness interventions for university students and personnel.^19,27^ Satisfaction with the online program was good and comparable to other studies evaluating online mindfulness training for the general public.^28^ As well, participants were satisfied with the modules’ content, length, and supporting materials, which may explain why two-thirds of participants completed all seven modules. This program completion rate is particularly important considering the very high attrition rates with online interventions in university students and young working adults,^19,28,29^ and research of in-person mindfulness programs amongst health care professionals that report attrition rates as high as 50%.^16^

While module completion was relatively high, regular daily practice with the meditation techniques was low. Other researchers have also found that adherence with meditation practice among university students who participated in an online mindfulness program was less than recommended.^28^ Suboptimal adherence with meditation practice amongst health care professionals attending in-person mindfulness training programs has also been reported. In a study of nursing students, only 12% established a daily meditation practice.^30^ Similarly, in a study of an abridged version of MBSR for first year medical students adherence to the home practice was low, despite students being enthusiastic about the program.^31^ Our previous work with a peer-led mindfulness program for medical students also showed that few participants established a daily meditation practice.^17^ Although we received limited feedback regarding challenges participants had establishing a regular meditation practice with the online program, time constraints, forgetting to practice, and stress were cited as reasons. The practice of mindfulness requires considerable commitment and improving adherence with meditation practice and determining the optimal “dose” and preferred type of practice (i.e., sitting meditation versus mindful yoga) are important issues to address in future research, as establishing a daily formal meditation practice is considered necessary to effect change in one’s capacity to self-regulate emotions and levels of mindfulness.^8^ For online programs, it would be worthwhile to investigate if the provision of support and encouragement through built-in email or text reminders can enhance practice adherence^32^ as offering support has been found to yield better outcomes and higher rates of adherence than pure self-help.^33^ Finding strategies to address systemic issues of time pressures in the medical curriculum is another important area to address if we expect medical students to be able to meaningfully integrate meditation into their everyday lives.

With respect to preliminary efficacy, no statistically significant improvement from baseline was noted for burnout. Although ratings of emotional exhaustion did improve in the expected direction, burnout rates remained unchanged from baseline, with about one in four students meeting threshold for burnout at both time points. These rates are comparable to other studies that have used the same criteria to classify medical students as burnt out.^34^ While it is possible that we would have detected a more robust effect of treatment on burnout if there was better compliance with the home meditation practice, no significant associations were noted between amount of meditation practice and pre-to-post study change in burnout scores. Our findings are in keeping with previous research that found no effect of mindfulness training on burnout measures. For example, Shapiro et al.^12^ showed that an eight-week in-person mindfulness intervention for health care professionals reduced perceived stress and enhanced self-compassion, but had no demonstrable effect on self-reported burnout. Further, a systematic review of mindfulness interventions for medical students concluded that mindfulness training does not reduce burnout, although it does show promise for other aspects of psychological health such as perceived stress.^35^ It has been suggested that the Maslach Burnout Inventory may not be sensitive enough to detect intervention effects because the scale asks respondents to count the frequency of a feeling as far back as a year.^36^ It is therefore possible that larger effects would have been obtained if we used additional measures of psychological well-being that exhibit better sensitivity to intervention effects. Alternatively, it is possible that the program itself may have impacted the way participants attended to their own inner experiences. As such, asking participants to retrorespectively report pre-program levels of distress after the program could potentially be an avenue to address this methodological limitation.

With respect to empathy, our participants had average baseline levels of empathy, with few participants meeting threshold for low empathy. We found no significant effect of our online intervention on empathy levels, although there was an increase in the expected direction. Our results concur with previous studies that found no significant effect of in-person mindfulness training on medical student empathy^17,37,38^ although positive effects have also been reported.^12,39^ The lack of statistically significant changes in empathy may be attributed, in part, to properties of the JES-S. Although this scale is widely used as a measure of physician empathy, recent work suggests that the JES-S mostly measure attitudes towards empathy rather than empathy.^40,41^ Moreover, the extent to which the JES-S measures empathic behaviour is controversial.^40^ As empathy is a complex and multifaceted construct, more nuanced effects of our online mindfulness program may have emerged if additional measures of empathy was employed.

We did find that the online intervention increased two facets of mindfulness (observe and describe) and self-compassion. The positive effect on self-compassion is not surprising considering the content of some of the modules focused on self-compassion, perfectionism, and self-acceptance. It remains unclear however what accounted for improvement in these outcomes as there was little correlation between adherence with the modules and meditation practice and outcome. A review of the mindfulness homework literature by Vettese and colleagues^42^ found that almost 50% of studies of in-person mindfulness-based programs failed to detect a significant relationship between the amount of meditation practice and study outcomes, suggesting that other aspects of mindfulness programs, such as group cohesion, may be important. It is conceivable that in the current study, the psychoeducational aspect of the modules or other factors accounted for the changes. Incorporating improved tools to measure adherence to a regular mindfulness practice in online programs for medical students and assessing the quality of the meditation practice, rather than frequency alone, may help elucidate the moderating effect of practice on outcome.

There are study limitations to note. First, the small sample size, compounded by the sub-optimal number of participants who completed post-study assessments, reduced power to find stronger effects regarding effectiveness and limits the generalizability of findings to other medical students. Second, the participants were predominantly female medical students, although this is in keeping with gender ratios reported in the literature.^19^ Third, the study employed an uncontrolled design and we cannot be certain that changes in aspects of mindfulness and self-compassion were due to the online intervention or to the passage of time or other events in the student’s clinical or personal experiences. Fourth, as noted above, our measures of psychological wellbeing and empathy may not have been sufficiently sensitive to detect expected effects. Fifth, we cannot rule out the possibility that positive evaluations of the program modules and satisfaction ratings were biased by social desirability. Finally, students who volunteered to participate in the study represent a self-selected sample that was more receptive to mindfulness training or in greater need of a stress reduction program. It will be important for future research to address these limitations.

Despite study limitations, this preliminary feasibility study suggests that an online mindfulness intervention is acceptable to medical students and may enhance aspects of mindfulness and self-compassion, and provides a solid basis for further research and development of online mindfulness programs for medical students. In keeping with the recommendations of a recent review, future research involving mindfulness interventions would benefit from pairing these programs alongside structural changes to optimize the likelihood of making an impact on levels of medical student distress^43^. Large randomized controlled trials are needed to provide a more definitive evaluation of the usefulness of an online meditation program for medical student wellness and future studies could utilize existing technologies available in mindfulness apps to better track meditation adherence and assess the relationship between practice adherence and outcome. Future research would also benefit from additional qualitative analysis to explore the benefits and shortcomings of an online mindfulness intervention and factors that contribute to poor adherence with meditation practice and attrition. Finally, it would be worthwhile to conduct comparative trials of in-person versus online mindfulness meditation programs to further establish the benefits of on-line programs for medical students and identify the unique impacts of in-person mindfulness training, including the opportunity of sharing and discussing experiences and the support of a group.
